# Are we “preparing” radiopharmaceuticals?

**DOI:** 10.1186/s41181-016-0011-7

**Published:** 2016-07-22

**Authors:** Clemens Decristoforo, Marianne Patt

**Affiliations:** 1 Department of Nuclear Medicine, Medical University Innsbruck, Innsbruck, Austria; 2 Klinik und Poliklinik für Nuklearmedizin, University of Leipzig, Leipzig, Germany; 3 Universitätsklinik für Nuklearmedizin, Medizinische Universität Innsbruck, Anichstr. 35, A-6020 Innsbruck, Austria

Today drugs are almost exclusively manufactured industrially. This has been driven by cost savings, but also by a public mandate of high quality and especially standardization, that is difficult to maintain in a non-industrial setting. On the other hand the same public asks for “personalized”or “precision medicine”(Reza Mirnezami et al. 2012) precisely tailored to each patient’s requirements. The more personalized medicines becomes, the less effective a large industrial production can be. In such a situation, not only drug manufacturers have to adopt their strategies, but also the regulatory framework, in which medicine is embedded and drugs are produced, has to implement these trends. That also holds true for radiopharmaceuticals (Lange et al. 2015).

Stimulated by activities especially of the EANM and its Radiopharmacy Committee, which drafted a number of guidelines and guidance documents (e.g. (Elsinga et al. 2010; Aerts et al. 2014)), recently PIC/S has released specific guidelines for radiopharmaceuticals prepared in healthcare establishments (Pharmaceutical Inspection Co-operation Scheme. Document PE 010–4 and Annex 3: Good practices for the preparation of radiopharmaceuticals in healthcare establishments 2014) and the EDQM has published a chapter on the “Extemporaneous Preparation of Radiopharmaceuticals” (Extemporaneous preparation of radiopharmaceutical preparations. Chapter 5.19). In these documents a clear distinction is made between industrial and small scale extemporaneous preparation of radiopharmaceuticals, providing guidance on “Good Practices” distinct from industrial standards.

The European Parliament and the European Commission now recently have issued a legal act that regulates the requirements for clinical trials within the European Union (EU regulation No 536/2014). The overall purpose of this regulation was to facilitate and harmonise clinical research in the member states and therefore the regulation has very clearly exempted the preparation of radiopharmaceuticals from the requirements of GMP (Decristoforo et al. 2014) if it “is carried out in hospitals, health centres or clinics, by pharmacists or other persons legally authorised in the Member State concerned to carry out such process, and if the investigational medicinal products are intended to be used exclusively in hospitals, health centres or clinics taking part in the same clinical trial in the same Member State”, thereby specifying the framework in which it should be applied, not being intended for radiopharmaceuticals manufactured industrially and being distributed. In a similar way a number of European member states have set up a regulatory framework in which radiopharmaceuticals for routine use (i.e. not for clinical trials) can be prepared on site without the requirements of a marketing authorisation. These exemptions can be derived from the definitions in Article 3 of Directive 2001/83 (The European Parliament and the Council of the European Union 2001), the so called magistral and officinal formulae.

In this context it should be considered that the European Court of justice has recently clarified the situation for “magistral formulae” defined as “any medicinal product prepared in a pharmacy in accordance with a medical prescription for an individual patient” (The European Court of Justice. Document 62013CJ0544 and Judgment of the Court (Third Chamber) of 16 July 2015). The European Court of Justice specified that “[such a preparation] must of necessity be prepared on the basis of a prior prescription issued by a professional person qualified to do so”. This prescription must, in addition “be ‘for an individual patient’” and “that patient must be identified before the medicinal product is produced and it must be produced specifically for that patient”. In a simpler wording such preparations have to be “extemporaneously”. It was also stated that “that the exception provided for in that provision can only concern situations in which the doctor considers that the state of health of his individual patients requires that a medicinal product be administered for which there is no authorised equivalent on the national market or which is unavailable on that market”, clearly excluding competition with licensed medicinal products. Also specifically excluded in this ruling were preparations “on the basis of the needs known in advance, to be used in emergency departments and, in any event, on the basis of orders placed before a specified patient had been identified” and “Preparations prepared and delivered to non-hospital pharmacies, on the basis of a ‘subscription’, even if an ‘initial medical prescription’ was drawn up for each specific patient”. The Court ruled that the aforementioned preparations were not “magistral” but medicinal products “prepared industrially or manufactured by a method involving an industrial process….. Such a process is characterised in general by a succession of operations, which may, in particular, be mechanical or chemical, in order to obtain a significant quantity of a standardised product.”

Translated to the preparation of PET radiopharmaceuticals it could be derived from this ruling, that PET preparations can be considered in many situations as being “magistral”. Typically a type of prescriptions by the physician referring an individual patient to Nuclear Medicine is provided, for whom the PET radiopharmaceutical is prepared, possibly with the exemption of FDG production in large departments where the preparation is made without necessarily always knowing the individual patient, and especially if it is shipped to different sites. However in this context also “officinal preparations” could be considered, in case a monograph for the radiopharmaceutical is available (which today is the case for most routinely used PET radiopharmaceuticals).

At the bottom line the discussion focusses on the question, are we “preparing” or “manufacturing” radiopharmaceuticals. If they are (extemporaneously, on a small scale, locally) prepared, exemptions as stated above may apply, whereas in they are (industrially) manufactured, the framework of GMP implemented within a manufacturing authorisation should be applied. The European Pharmacopeia (Ph. Eur.) as a legal basis for almost all European countries have tried to clarify what they see as “preparation”, in the monograph on “Pharmaceutical Preparations” (Pharmaceutical preparations. Monograph N° 2619) the following definition can be found: “the ‘manufacture’ of unlicensed pharmaceutical preparations in pharmacies or other healthcare establishments (the term ‘preparation’ is used instead of ‘manufacture’ in order clearly to distinguish it from the industrial manufacture of licensed pharmaceutical preparations)”. Even more specifically the term radiopharmaceutical preparation is used in the General text 5.19 of the Ph. Eur. and explicitly includes kit-based preparations as well as unlicensed preparations for PET and SPECT.

Applying this definition to radiopharmaceuticals underlines the clear distinction between the industrial manufacturing in a framework of “licensing” i.e. marketing authorisation or clinical trials aiming at a marketing authorisation versus the local, extemporaneous preparation of radiopharmaceuticals being it for patient diagnosis or within the framework of a local clinical trial.

Discussions with regulatory bodies should try to stress this point, giving the Nuclear Medicine community the possibility to provide required (novel or established) radiopharmaceuticals for patients needs and to bring the speciality forward towards the times of personalized medicine, tailor-made for patients need that deserve the best medicines, being radioactive or not.

## Glossary

EANM: European Association of Nuclear Medicine
EDQM: European Directorate for the Quality of Medicines & HealthCare
Extemporaneous: individually (prepared) for a specific patient or patient group, supplied after preparation (from European Pharmacopoeia: Pharmaceutical Preparation)
GMP: Good Manufacturing Practice
Kit: Any preparation to be reconstituted or combined with radionuclides in the final radiopharmaceutical, usually prior to its administration.
Magistral: prepared in a pharmacy in accordance with a medical prescription for an individual patient
Manufacture: larger scale commercial/industrial production; all operations of purchase of material and products, Production, Quality Control, release, storage, distribution of medicinal products and the related control (from European Pharmacopoeia: Pharmaceutical Preparations)
Officinal: prepared in a pharmacy in accordance with the prescriptions of a pharmacopoeia and intended to be supplied directly to the patients served by the pharmacy in question
Parenteral: sterile preparations intended for administration by injection, infusion or implantation into the human or animal body
PET: Positron Emission Tomography
PIC/S: Pharmaceutical Inspection Convention and Pharmaceutical Inspection Co-operation Scheme
Preparation: small scale non-industrial production; the ‘manufacture’ of unlicensed pharmaceutical preparations in pharmacies or other healthcare establishments (from European Pharmacopoeia: Pharmaceutical Preparation)
Radiopharmaceutical: Any medicinal product which, when ready for use, contains one or more radionuclides (radioactive isotopes) included for a medicinal purpose.
